# Near-Infrared Fluorescent Nanoprobes for *in Vivo* Optical Imaging

**DOI:** 10.3390/nano2020092

**Published:** 2012-03-30

**Authors:** Chai-Hoon Quek, Kam W. Leong

**Affiliations:** 1 Department of Mechanical Engineering and Materials Science, Duke University, Durham, NC 27708, USA; Email: cq4@duke.edu; 2 Department of Biomedical Engineering, Duke University, Durham, NC 27708, USA

**Keywords:** near-infrared, *in vivo* imaging, fluorescent dyes, nanotechnology, nanoprobes

## Abstract

Near-infrared (NIR) fluorescent probes offer advantages of high photon penetration, reduced light scattering and minimal autofluorescence from living tissues, rendering them valuable for noninvasive mapping of molecular events, assessment of therapeutic efficacy, and monitoring of disease progression in animal models. This review provides an overview of the recent development of the design and optical property of the different classes of NIR fluorescent nanoprobes associated with *in vivo* imaging applications.

## 1. Introduction

The first medical imaging was realized in the late 1895 by Wilhelm Röntgen shortly after he discovered X-ray and applied to capturing the images of the bones of a hand on film [1]. The immediate consequence of this discovery triggered intense development of new imaging technologies, such as X-ray computed tomography, magnetic resonance imaging, positron emission tomography, ultrasound and optical imaging, that are indispensable to diagnostic medicine (Table 1) [2,3]. These imaging technologies differ predominantly in the following aspects: resolution, penetration depth, temporal resolution and energy expended for generation of the image. 

**Figure 1:**
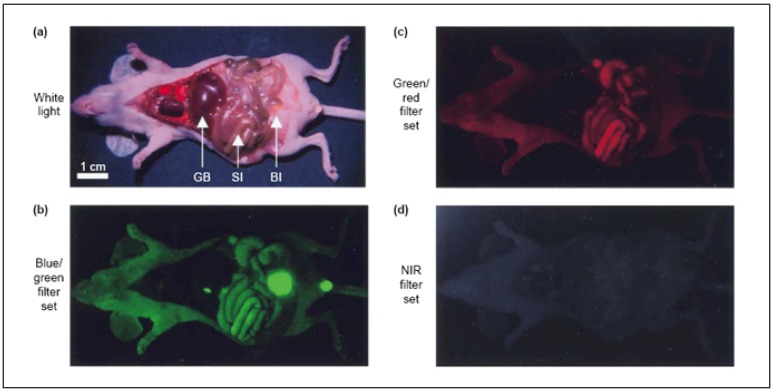
Wavelength-dependent autofluorescence of vital organs and body fluids. (**a**) Image of the viscera of an athymic nude mouse taken immediately after sacrifice. The arrows indicate the location of gall bladder (GB), small intestine (SI) and bladder (Bl). Tissue autofluorescence was imaged using three different excitation/emission filter sets; (**b**) blue/green (460–500 nm/505–560 nm); (**c**) green/red (525–555 nm/590–650 nm); and (**d**) Near-infrared (NIR) (725–775 nm/790–830 nm). (Reprinted with permission from [14], copyright 2003 Elsevier).

**Figure 2:**
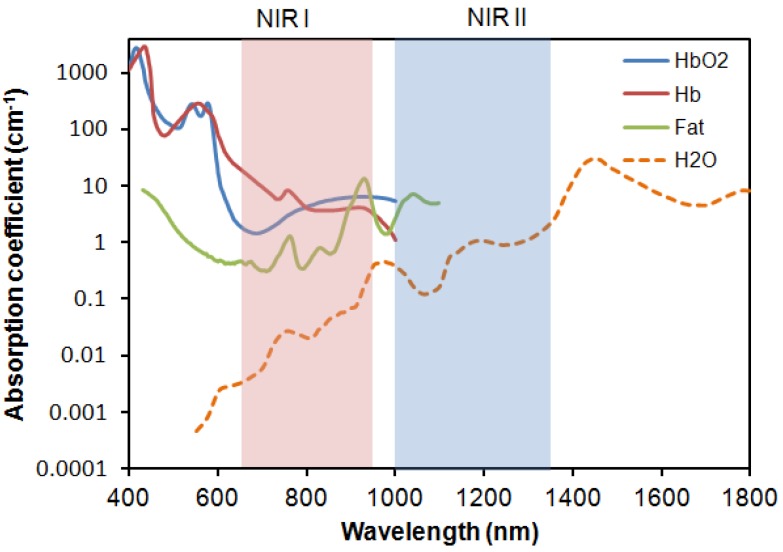
Absorption coefficient (on a log scale) of oxygenated blood, deoxygenated blood, fatty tissue and water as a function of wavelength.

Today, these modern imaging technologies coupled with newly developed imaging probes are widely used in monitoring disease progression, interrogating cellular and molecular events, evaluating safety and toxicology in drug discovery and development, and assessing therapeutic efficacy *in vivo* [2,4,5,6,7,8,9,10,11,12]. Among the different imaging modalities, optical imaging, which owes its origin to single-cell *in vitro* studies, is attractive for small animal imaging because of its lower cost, portability, and potentially high spatial resolution with customizable fine-tuning. Despite being one of the most attractive techniques to provide noninvasive and nonionizing *in vivo* visualization [13], optical imaging is impeded by the tendency of living biological tissues to absorb and scatter photons and generate strong autofluorescence, which interferes with signal collection and processing (Figure 1) [14]. In addition, living tissues also contain other major NIR absorbers, such as water, lipids oxyhemoglobin and deoxyhemoglobin [5] that prove challenging for optical imaging. To overcome these barriers, intense research has focused on developing highly sensitive and efficient fluorescent probes that function in the biologically transparent window of the first and second NIR region (NIR I, 650–950 nm, and NIR II, 1000–1350 nm) (Figure 2) [15]. This review will provide a broad overview of the available NIR fluorescent probes, their optical properties and potential applications for *in vivo* imaging.

**Table 1:** Overview of imaging systems for small animals.

Modality	Resolution	Depth	Optimal use	Signal	Training/ expertise required	Cost ^+^
MRI	10–100 μm	No limit	Anatomical assessment, investigation of physiological, metabolic, molecular and genetic events.	RF ^*^ waves (Nonionizing radiation)	Yes (Certified radiologists)	$$$
PET	0.8–1.4 mm	No limit	Investigation of physiological, metabolic, molecular and genetic events.	γ-rays (Ionizing radiation)	Yes (Certified radiologists)	$$$
SPECT	0.8–1.4 mm	No limit	Investigation of physiological, metabolic, molecular and genetic events.	γ-rays (Ionizing radiation)	Yes (Certified radiologists)	$$
CT	50 μm	No limit	Anatomical assessment.	X-ray (Ionizing radiation)	Yes (Certified radiologists)	$$
Ultrasound	50 μm	mm	Anatomical assessment, investigation of physiological, metabolic, molecular and genetic events.	Sound waves (Nonionizing radiation)	Yes (Certified sonographers)	$$
Fluorescence optical imaging	0.3 µm	<1 cm	Metabolic, molecular and genetic events.	Light waves (Nonionizing radiation)	No	$

MRI, Magnetic resonance imaging; PET, Positron emission tomography; SPECT, Single photon emission computed tomography; CT, Computed tomography. ^∗^ RF, radiofrequency, ^+^ Cost of system: $ < 100,000; $$ 100–300,000; $$$ 1–3 millions.

## 2. Labeling Mechanism of Fluorescent Probes

**Figure 3:**
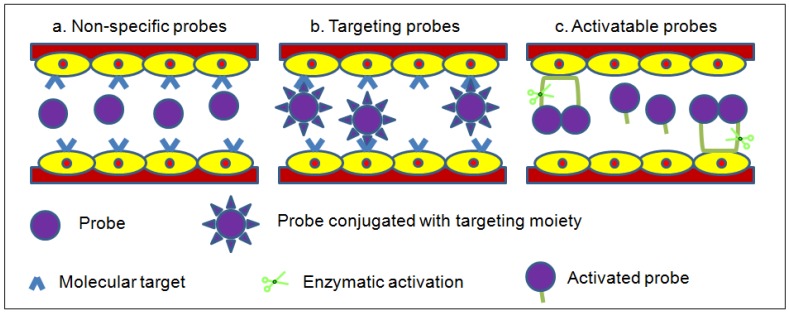
Modes of contrast generation of fluorescent probes. (**a**) Non-specific compartmental distribution of the probes. (**b**) Targeted binding of probes via surface ligands to molecular targets. (**c**) Activatable probes quenched in their native state and become fluorescent by enzyme-mediated cleavage.

Optical imaging has increasingly been used for dynamic noninvasive imaging of biological events in mouse models. Due to the lack of NIR fluorescence contrast generated by most tissues in the biological transparency window, exogenous fluorescent probes have to be administered for *in vivo* studies in order to visualize living tissues in its native physiological state. Ideally, the fluorescent probes to be administered should be biologically stable in the *in vivo* environment, and accumulate and produce imaging contrast at the target site. Fluorescent probes are classified according to their mechanism of contrast generation, collectively as non-specific, targeting and activatable (Figure 3) [6]. 

Non-specific probes simply have differential distribution and are used to assess physiological processes such as changes in blood volume, permeability and perfusion in angiogenesis (Figure 3a). Typically, they achieve only low target-to-background signals due to the non-binding circulating probes producing significant background fluorescence within the compartment. 

Tissue- or cell-specific contrast is created by coupling a targeting moiety to a fluorescent probe that binds specifically to a receptor thus generating the readout signal (Figure 3b). These probes can report more detailed information about the biological events than non-specific probes. Targeting probes can achieve high target-to-background signals provided the targeting moiety has a high affinity for the receptor and any unbound probes are thoroughly removed from the system, thereby reducing the background fluorescence. 

Activatable probes comprise of donor-acceptor fluorophores that are coupled to each other in close proximity to maintain a quenched state (Figure 3c). The fluorescence emission is activated by enzyme-mediated cleavage that releases the fluorescent probes. Activatable probes can attain high target-to-background signals as these probes in their native injected state are relatively undetectable.

## 3. Small Organic Fluorophores

### 3.1. Non-Specific Organic-Dye Probes

**Figure 4:**
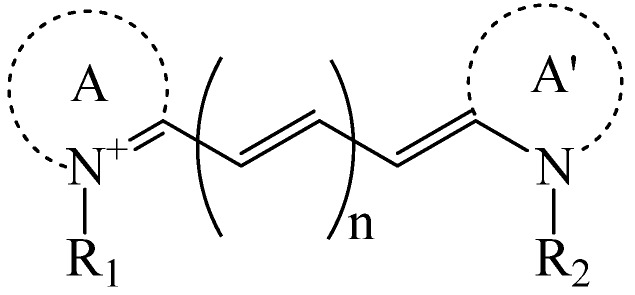
Generalized structure of cyanine dyes. A and A' are two quaternized heteroaromatic bases.

The choice of a suitable dye for *in vivo* imaging depends on many factors. The most important consideration would be the molar extinction coefficient and quantum yield of the dye in the NIR region [16,17]. Of all the dyes, cyanine makes up the majority of commercial fluorescent probes for *in vivo* applications. Cyanine is a synthetic dye family of the polymethine group with a conjugated chain of odd number of carbon atoms linked between two nitrogen centers (Figure 4) [18]. Among this class of compounds, carbocyanine dyes with indolic groups are readily available commercially and have also been synthesized in a large variety of analogues. The structure of carbocyanine is formed by reaction of two indolic isomers (either identical or different) linked on each end of a C1, C3 or C5 methine. The absorption and fluorescence emission wavelength is determined both by the chain length of the methine and the side chains attached to the indolic groups. Table 2 shows a series of commercially available carbocyanine dyes emitting in the visible to NIR range. These dyes generally exhibit high molar extinction coefficients but only have moderate fluorescence quantum yields up to 30%. A prominent representative of the NIR cyanine dye is Indocyanine green (ICG). ICG was first synthesized in the fifties [19] and is the only clinically approved dye available commercially. It has been used in optical imaging of changes in blood-brain barrier permeability after thrombus formation in a mouse model of cerebral venous thrombosis [20] and liver perfusion in mouse [21]. Clinically, ICG has been used in clinical retinal angiography [22], hepatic function testing [23] and imaging of human brain [24,25]. Since ICG binds tightly to plasma proteins and becomes confined to the vascular system, they are widely used for intraoperative assessment of vascular flow in cardiovascular surgery [26,27,28]. Because of its amphophilicity and a shortage of functional groups available for conjugation, ICG could only function as a non-specific probe for *in vivo* imaging. 

### 3.2. Targeting Organic-Dye Probes

Covalent attachment of targeting moieties such as antibodies, antibody fragments, proteins and peptides to fluorescent dyes can significantly improve the target-to-background signal. The strategy of conjugating antibodies to cyanine dyes were first demonstrated by Folli *et al.* and Ballou *et al.* [29,30] and applied to fluorescence imaging of tumor in mice. Antibodies, however, have found limited utility due to their unfavorable pharmacokinetics. The typical circulating half-life of antibodies is much shorter than the time required to access a small tumor with reasonable accumulation, thus rendering effective targeting rare using antibodies [31]. 

To improve the pharmacokinetics of *in vivo* contrast agents, a possible approach is to reduce the conjugate molecular size while still preserving the targeting affinity of the labeled dyes. Neri *et al.* demonstrated this idea by conjugating antibody single chain fragments selected from phage display libraries against an angiogenesis-associated oncofetal fibronectin isoform to cyanine dyes and applied to imaging of angiogenesis in a variety of animal models [32,33]. Alternative strategy is also shown in the work by Achilefu *et al.* and Licha *et al.* highlighting the successful application of receptor-specific peptide-dye conjugates for fluorescent imaging of tumors [34,35,36]. As a whole, fluorescent dyes can be readily functionalized to achieve targeting.

**Table 2:** Structures and optical characteristics of commercial NIR cyanine dyes.

Dye	Structure	Abs/Em (nm)	Molar extinction coefficient (M^−1^ cm^−1^)	Quantum yield
Indo-Cyanine Green (ICG) [17]	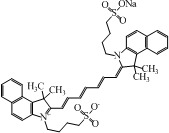	807/822	121,000	0.09
Cy5.5 NHS ester ∗	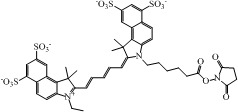	675/694	250,000	0.23
Cy7 NHS ester ∗	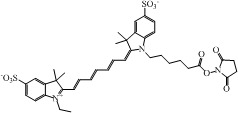	747/774	200,000	0.28
Cy7.5 NHS ester ^§^	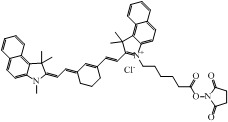	788/808	223,000	N.A.

∗ Information is obtained from supplier GE Healthcare website; ^§ ^Information is obtained from supplier Lumiprobe website.

### 3.3. Activatable Organic-Dye Probes

The concept of activatable NIR probes was introduced by Weissleder *et al.* for *in vivo* imaging of enzyme activity [37]. Enzyme-activatable probes contain either two identical or different fluorophores linked in close proximity to each other by a specific peptide linker. The fluorescence of the probes is essentially undetectable in the quenched state. After enzymatic cleavage, the fluorophores are separated to restore the fluorescence emission. A large number of activatable probes have been documented, among them the enzyme activation of NIR targeting dyes is mediated mainly by tumor associated proteases, such as cathepsins, caspases and matrix metalloproteinases [38,39,40,41,42,43]. The ability of activatable fluorescent probes to detect gene expression is particularly useful as a means to diagnose malignant molecular process in the early disease state [44]. 

## 4. Nanomaterial-Based Fluorescent Probes

Despite the long history of *in vivo* optical imaging, organic dyes have suffered from small Stokes shifts, poor photostability, high plasma protein binding rate, aggregation and background fluorescence in aqueous medium [45]. Attention has been increasingly channeled toward nanotechnology to search for new class of more effective probes. Many nanomaterials are already widely used as catalysts, and energy storage and electronic devices [46,47,48,49,50]. Their application to the biomedical arena has attracted a similarly enthusiastic following. Interest in the nanomaterials arises from the fact that at nanoscale the properties of materials can be very different from their bulk counterparts. Firstly, nanomaterials when compared to the same mass of material existing in a larger form have a much higher surface area. As such, nanomaterials are more chemically reactive because of the high surface energy. In addition, at nanoscale range, quantum effects begin to dominate the behavior of matter, affecting the optical, electrical and magnetic behavior of materials [51,52]. 

Lately, the integration of nanotechnology with medicine has found many novel applications of nanomaterials. Nanoparticles can be engineered to overcome biological barriers for effective and targeted delivery of drugs, genes, and contrast agents [53]. Active moieties such as proteins, peptides and nucleic acids can easily be conjugated to the surface of the nanoparticles for use as non-specific, targeting or activatable nanoprobes. The following sections are devoted to the recent development of some particulate NIR fluorescent nanoprobes for potential *in vivo* imaging. 

### 4.1. Quantum Dots

**Figure 5:**
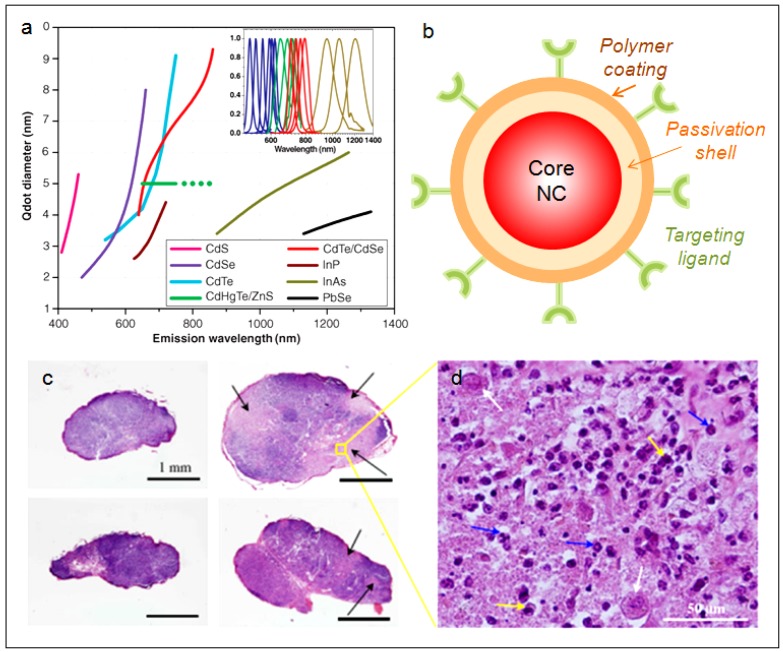
(**a**) Emission maxima and sizes of quantum dots of different composition. (Reprinted with permission from [54], copyright 2005 Science). (**b**) Typical structure of a quantum dots (QD) for biomedical applications. The core of the QD is passivated by a second semiconductor material. The core/shell QD is made hydrophilic and biocompatible by a polymer coating. The surface of the QD is conjugated with a targeting ligand for specific recognition and interaction with biological molecules. (**c**) Histology sections of lymph node (LN) 10 days post-injection: control regional auxiliary LN (RALN) (upper left), control right lateral thoracic LN (RLTLN) (lower left), RALN injected with QDs (upper right), and RLTLN injected with QDs (lower right). The lighter regions indicated with black arrows are the sites of inflammation. (**d**) Magnification of an inflammation area from RALN. There were many polynuclears observed but only some were indicated with blue arrows. Histiocytes (yellow arrows) and also vacuoles of digestion were also observed. (Reprinted with permission from [95], copyright 2009 Academy of Molecular Imaging).

Fundamentally, quantum dots (QDs) are semiconductor nanocrystals that absorb photons of light and re-emit photons at a different wavelength (Figure 5a) [54]. Typically, QDs are of ~2–20 nm in diameter depending on the core composition and the surface coating or functionalization. Most of the QDs reported have a core/shell structure (Figure 5b) with the core composed of atoms from periodic groups II–VI (CdSe, CdTe, CdS, PbSe, ZnS and ZnSe), III–V (GaAs, GaN, InP, InAs), and IV–VI (PbS). QDs differ from traditional organic fluorescent dyes and naturally fluorescent proteins in several important aspects. Firstly, QDs are extremely efficient in generating fluorescence, often by an order of magnitude higher than traditional fluorophores. In addition, QDs fluoresce without involving conjugated double-bond systems, thus exhibit greater photostability to enable long-term imaging without the concern of photo-induced deterioration. Another practical advantage of QDs is that their emission spectra are narrow and symmetric, thus minimizing any overlapping of colors in multi-component imaging applications. With the same underlying material, varying the size of the QDs could give rise to different emission peaks, giving QDs unprecedented tunability. This enables the use of a single laser to excite and achieve multicolor emission for multiplexed assays [53,55,56,57]. These unique properties of QDs have been increasingly exploited as bioimaging agents [58,59,60,61], theranostic platforms to deliver and track siRNA-based therapy [62,63,64,65], nanosensors to study intracellular trafficking and unpacking of DNA nanocomplexes [66,67,68,69], and cell lineage-tracing agents to follow the development of stem and progenitor cells *in vivo* developmental [70,71]. 

For *in vivo* imaging, the fluorescent emission wavelengths of the QDs ideally should be around 700–1000 nm, in the NIR region to minimize the endogenous fluorescence and the interference from major absorbers in the body. As presented in Figure 5a, CdTe [72,73], CdTe/CdSe [74], CdHgTe/ZnS [75,76], InAs [77], InAs_x_P_1−x_/InP/ZnSe [78], and PbSe [79,80] are the possible QDs that emit in the NIR I region. All these QDs are usually passivated with a layer of ZnS or ZnSe to protect the core from oxidation and to prevent the leaching of toxic heavy metal ions such as Cd, Hg, As and Pb into the surrounding solution. However, with the several reports indicating Cd-containing QDs showing cytotoxicity under extreme conditions [81,82,83,84,85], there is a rising concern of using them for long term *in vivo* applications. Therefore, attention has been channeled to develop new class of quantum dots comprising of less-toxic elements. Some of these newly developed NIR QDs include Cu-doped InP/ZnSe [86], CuInSe [87,88], and CuInS_2_/ZnS [12,89,90].

Among the synthetic routes of QDs, the most commonly used technique is to first generate the core by injecting liquid precursors into nonpolar coordinating organic solvent at a temperature as high as 300 °C and subsequently growing the shell layer of ZnS onto the core [54,56,91,92]. QDs generated by this method are by default hydrophobic and are unsuitable for use in biological systems. To render them dispersible in aqueous medium, the QDs such as CuInS_2_/ZnS are transferred into aqueous phase either by ligand exchange with dihydrolipoic acid/polyethylene glycol 1000 (DHLAPEG100) or by encapsulation in phospholipid micelles [60,90,93,94]. The resulting water-soluble QDs emit at wavelength greater than 700 nm and could achieve good quantum yield of 30%. *In vivo* sentinel lymph node imaging with the CuInS_2_/ZnS QDs were performed in the work of Pons *et al.* by subcutaneous injection into regional lymph nodes (LN) of healthy mice. It was observed that the QDs preferentially accumulated at the injection site, in the two regional LNs and to a lesser extent in other organs over a 10-day observation period [95]. The results suggest that the LNs would be more sensitive toward acute QD toxicity. They further compared the inflammatory response of the two regional LNs treated with CuInS_2_/ZnS and CdTeSe/CdZnS QDs. Histological sections indicated inflammation only occurred at 10 times dose concentration for cadmium-free QDs than for the Cd-containing counterparts (Figure 5c,d). This study highlights the advantage of cadmium-free QDs for safer NIR *in vivo* imaging.

### 4.2. Colloidal Silicon Quantum Dots

Silicon is one element that is most widely used in the microelectronics industry due to its outstanding performance as an electronic material [96,97]. Its inherit nontoxic and environmentally friendly characteristic has attracted much attention to develop silicon quantum dots (Si QDs) for biological applications [98,99,100,101]. An important step toward realizing these applications is the fabrication of colloidally and optically stable, water-dispersible Si QDs. Silicon QDs are typically prepared in a two-step process: first by CO_2_ laser pyrolysis of silane to generate non-photoluminescent crystalline Si particles with an average diameter of 5 nm [102]. Etching with mixture of hydrofluoric acid and nitric acid is then followed to reduce the size and passivate the surface of the particles [103]. Using this method Si QDs with size ranging from 1 to 5 nm and emission from blue to NIR can be obtained. 

**Figure 6:**
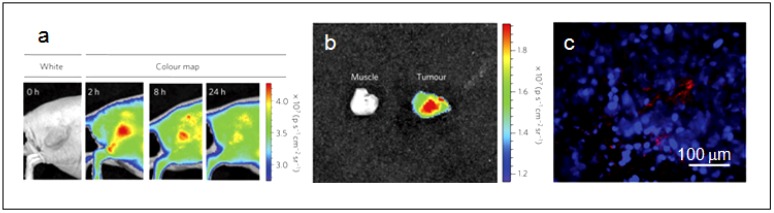
(**a**) Representative fluorescence images of a mouse with an MDA-MB-435 tumor. The mouse was imaged using a 615–665 nm excitation filter and an 810–875 nm emission filter at the indicated time post-intravenous injection of D-LPSiNPs (20 mg/kg). A strong signal from D-LPSiNPs is observed in the tumor, indicating significant passive accumulation in the tumor by the enhanced permeability and retention (EPR) effect. (**b**) *Ex vivo* fluorescence images of the tumor from the mouse used in (a). (**c**) Fluorescence images of a tumor slice from the mouse in (a). Red and blue indicate D-LPSiNPs and cell nuclei (DAPI stain). (Reprinted with permission from [99], copyright 2009 Macmillan).

In 2004, Li *et al.* first demonstrated the potential of Si QDs for biological applications. They grafted water-soluble polyacrylic acid Si QDs to label Chinese hamster ovary (CHO) cells for *in vitro* imaging [104]. In the recent work by Park *et al.* they delivered Si QDs loaded with anti-cancer drug doxorubicin D-LPSiNPs into BALB/c mice and monitored both accumulation and degradation *in vivo* (Figure 6) [99]. Si QDs are exhibiting the attractive attributes to be an effective and biocompatible nanomaterials system for theranostic purpose; they will undoubtedly see intense effort for further improvement and innovative applications.

### 4.3. Gold Nanoclusters

Fluorescence of noble metal nanoclusters (NCs) of gold and silver comprising of several to tens of atoms have drawn great attention in the past decade because of their remarkable optical properties [105,106,107,108,109,110,111]. Because of their minuscule size, high chemical stability and biocompatibility, NCs are increasingly being exploited for sensing [112,113,114], biolabeling [115,116] and bioimaging [117]. Typically, the size of NCs is less than 2 nm and their properties are governed by their subnanometer dimensions. Their size regime is intermediate of atomic and nanoparticles in which they no longer have plasmon resonance and Mie’s theory is non-applicable [111]. In fact, the size of noble metal NCs is comparable to the Fermi wavelength of the conduction electrons, leading to molecule-like features such as discrete size-dependent electronic transitions, fluorescence and charging properties. 

Multitudes of strategies have been developed for the synthesis of gold nanoclusters (AuNCs). One of which is chemical reduction of Au precursors in the presence of thiol stabilizers to afford AuNCs that fluoresce in the blue to near-IR regions [118,119,120,121,122]. However these NCs exhibit low quantum yields (QY) (0.001–0.1%). AuNCs can also be prepared via template-assisted synthesis within the cavities of dendrimers [111] with greater than 10% QY at the expense of fairly long reaction time of ~2 days along with the production of large nanoparticles (NPs) as byproduct. Another commonly used technique to prepare AuNCs is by ligand-induced core etching of metal NPs to yield blue-emission NCs [123]. Fine-tuning of the core etching method led Lin *et al.* to generate NIR AuNCs by first producing 6-nm diameter gold nanoparticles core stabilized with dihydrogenlipoic acid (DHLA) (AuNC@DHLA) [124,125]. Conjugation of AuNC@DHLA with biomolecules can easily be achieved with the carboxyl group on DHLA via carbodiimide chemistry. Polyethylene glycol (PEG, 5 kDa), PEG-biotin and avidin have been attached in this manner. The shortcoming of these NCs is the low quantum yield of 1–3%. Nevertheless, the authors demonstrated the specific labeling capability of the AuNCs toward fixed human hepatoma cells (HepG_2_) and also the non-specific uptake of the AuNCs by human aortic endothelial cells. The results make obvious that these AuNCs are relatively nontoxic. 

**Figure 7:**
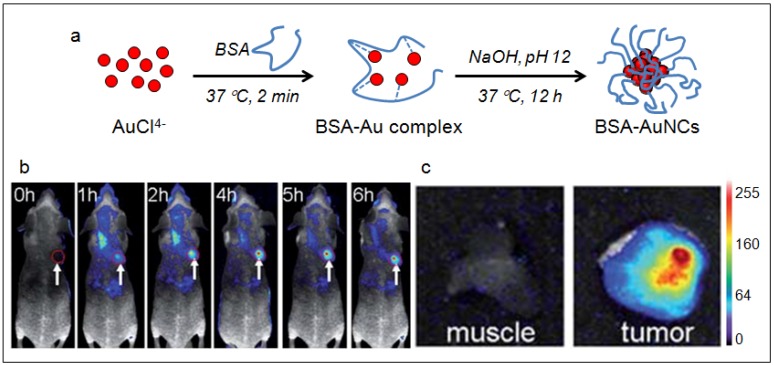
(**a**) Schematic of the formation of AuNCs in BSA solution. (**b**) Fluorescence images of mice with a MDS-MB-45 tumor. Strong signal from AuNCs was observed in the tumor (red circle), indicating significant passive accumulation in the tumor by the EPR effect. White arrows indicate tumor site. (**c**) *Ex vivo* fluorescence image of the muscle and tumor tissue around the tumor from the mice used in b. (Reprinted with permission from [128], copyright 2010 The Royal Society of Chemistry).

With increasing interest in applying NIR fluorescent probes for bioimaging, effort has been devoted to developing biocompatible AuNCs via aqueous synthetic routes with biological molecules [126]. Xie *et al.* [127] has developed an innovative method by exploiting the reducing capability of bovine serum albumin (BSA) to prepare AuNCs consisted of 25 gold atoms with red emission [127]. The synthesis is similar to the biomineralization activities of organisms. Once added to aqueous BSA solution the Au ions were sequestered and entrapped within the protein molecules. The reduction ability of BSA was activated when the pH of the reaction was adjusted to ~12 and the ions were progressive reduced to AuNCs *in situ* (Figure 7a). Additional reductant, such as NaBH_4_ would be required to yield AuNCs that emit at 683 nm. The quantum yield of the NIR AuNCs produced by this method was also low (0.1%). Despite that, Wu *et al.* demonstrated the applicability of these AuNCs for *in vivo* tumor fluorescence imaging using MSD-MB-45 and HeLa tumor xenograft models. They attributed the high accumulation of the NIR AuNCs in the tumor areas to the enhanced permeability and retention (EPR) effects (Figure 7b) [128]. 

## 5. Carbon Materials

### 5.1. Single-Walled Carbon Nanotubes

Carbon nanotubes (CNTs) represent another class of nanomaterials attract intense interest since their discovery in 1991 [129]. CNTs are cylindrical tubes of sp^2^ carbon with superior optical, electrical, and mechanical properties, thus have wide range of potential applications as nanoelectronics devices and engineered composite materials [48,49]. In addition, CNTs have been extensively explored as intracellular delivery vehicles for drugs, proteins and genes in cancer therapy [130,131].

CNTs are categorized as single-walled nanotubes (SWNTs) and multi-walled nanotubes (MWNTs). So far, only SWNTs have been reported to possess high emission in the NIR II region, making them suitable for deep-tissue in vivo imaging [132]. In general, SWNTs are most commonly synthesized by chemical vapor deposition that involves the heating of an alumina substrate with a layer of transition-metal catalytic nanoparticles, which serve as seeds to nucleate the growth of the nanotubes in a furnace with a stream of hydrocarbon gas flowing through the tube reactor [133]. SWNTs synthesized by this method have vast possibilities in the type of carbon tube “molecules”, giving rise to variation in band gaps ranging from ~10 meV to ~0.5 eV [133].

**Figure 8:**
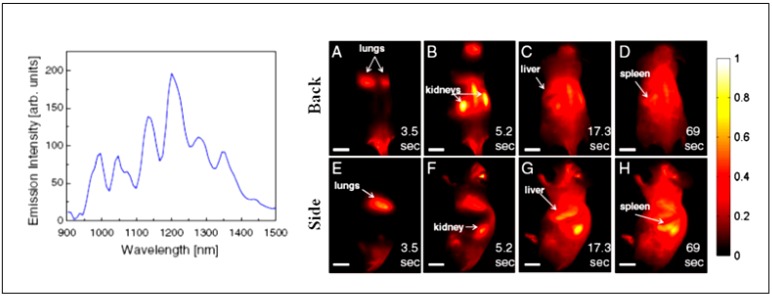
*Left panel*: Fluorescence spectrum of DSPE-mPEG functionalized SWNTs excited at 808 nm, showing several emission peaks in the NIR II ranging from 1000–1400 nm. *Right panel*: Frames from video imaging of mice injected with SWNTs. At 3.5 s (**A** and **E**) post tail-vein injection, the lungs are the dominant feature, corresponding to flow of oxygen-poor, SWNT-rich blood to the lungs. At 5.3 s (**B** and **F**), the SWNT-rich blood flows through the highly vascularized kidney followed by the liver at 17.3 s (**C** and **G**) and the spleen at 69 s (**D** and **H**). (Reprinted with permission from [134], copyright 2011 PNAS).

Welsher *et al.* suggested that the biggest obstacle in realizing the potential of SWNTs as NIR II probes is producing SWNTs with high quantum efficiency and good biocompatibility [132]. To overcome this issue, the authors came up with a strategy of first debundling and solubilizing the hydrophobic, pristine SWNTs in sodium cholate by sonification, followed by surfactant exchange to displace the sodium cholate with pegylated phospholipid (DSPE-mPEG). The resulting water-soluble SWNTs exhibit several emission peaks across the NIR II region when excited at 808 nm (Figure 8). When delivered into athymic nude mice via tail-vein injection, the systemic circulation and biodistribution of the SWNTs can be followed for over two minutes (Figure 8) [134]. This work shows the advantages of fluorescence imaging in the NIR II region and their potential impact in advancing nanomedicine. 

## 6. Conclusion and Outlook

The potential of NIR fluorescent nanoprobes for *in vivo* application is unlimited. The drawbacks of organic dyes such as poor photostability, undesired aggregation and fluorescence in aqueous solution can be resolved by innovative NIR nanoprobe designs. In particular the versatile, nanomaterials-based NIR fluorescent probes offer ample opportunity to optimize their optical and targeting properties. Among all nanomaterial-based fluorescent probes, Si QDs is the only system that can be directly loaded with drugs and biodegraded into silicic acid that can be excreted efficiently through renal clearance. Like other nanomaterial-based fluorescent probes, improvement of their quantum efficiency, however, will still require significant effort. Robust and environmentally friendly synthetic routes that can produce these nanomaterials-based NIR nanoprobes with uniform size and colloidal stability represent other significant challenges. The widespread application of NIR fluorescent nanoprobes awaits demonstration of their long term biocompatibility, *in vivo* targeting efficacy as well as parallel development of advanced optical instrumentation for deep-tissue imaging capability. Nevertheless, their role in advancing the field of nanomedicine will undoubtedly be essential. 
